# Three-Dimensional Human Gait Pattern: Reference Data for Young, Active Women Walking with Low, Preferred, and High Speeds

**DOI:** 10.1155/2019/9232430

**Published:** 2019-01-03

**Authors:** Slawomir Winiarski, Jadwiga Pietraszewska, Bogdan Pietraszewski

**Affiliations:** ^1^Biomechanics Division, University School of Physical Education in Wroclaw, Wroclaw 51-612, Poland; ^2^Department of Biology and Motor Sports Fundamentals, University School of Physical Education in Wroclaw, Wroclaw 51-612, Poland

## Abstract

Normal gait pattern is the key component in the investigation of pathological gait patterns. In computer motion analysis there is a need to include data from participants with different somatic structures to develop a normative database or to limit the database results to a specific population. The aim of this study was to determine kinematic gait patterns for young, active women walking with low, preferred, and self-selected speeds with regard to their somatic characteristics. Laboratory-based gait analysis was performed on 1320 gait cycles taken from 20 young, active women walking with three different speeds. Comprehensive anthropometric measurements and descriptive statistics were used to describe spatiotemporal and angular variables at each walking condition. The results demonstrated some significant differences in young, active women walking between different speeds and compared to the literature. This suggests that there is a need to include data from participants with different somatic structures to develop a normative database or limit the database results to a specific population. Detailed linear and angular kinematic variables allow for proper adjustment of parameters depending on the gait speed of people with locomotion disorders.

## 1. Introduction

It is a known fact that while walking calmly people select a particular walking speed. Preferred walking speed (PWS) is a speed similar to walking at speeds that minimise the energy expenditure per unit distance [1–3]. The walking speed, different than PWS, significantly affects kinematic and kinetic gait patterns [1, 4]. As have been reported in clinical trials, low walking speeds can be related to limited movements and joint kinetics. High walking speeds can lead to excessive joint loading and musculoskeletal system overload [5]. Clinicians need reliable reference data to assess these gait abnormalities and methods for patients to use to prepare themselves for physiotherapy. Gait parameter analysis can be used to determine the efficiency of rehabilitation, conservative management, or surgical treatment (i.e., cerebral palsy, muscular dystrophy, or Parkinson's disease) [6, 7].

Increased application of gait analysis increases the need for understanding how walking speed and somatic variables affect the gait pattern. Adult women often show a slight dominance of endomorphy, with little of the other components or their balanced participation. This may directly lead to different functional aspects [8]. Previous studies of female gait have almost exclusively focused on reporting gait variables for a well-defined research group. The following somatic parameters were often described: age, sex, body mass, and height. Al-Obaidi et al. [9] compared gait values of 15 males and 15 females from Kuwait with the results obtained by Oberg et al. [10, 11] for matched Swedish subjects. Women in Kuwait walked slightly faster than their Swedish peers; cadence was higher for Swedish men walking at PWS and women walking at high speed. To develop a normative database, the authors decided to include data on diverse somatic builds. Forczek and Staszkiewicz [12] and Bertuit et al. [13] reported reference data for healthy women in the study of pregnant women, while da Silva-Hamu et al. [14] tested the effect of obesity on kinematics and gait parameters in young women. Fryzowicz et al. [15] presented reference data on spatiotemporal parameters, joint angles, ground reaction forces, and plantar pressures for 28 young, healthy women at the age of 21. Hollman et al. [16] performed a factor analysis on 294 participants with almost 20 spatiotemporal gait variables and created normative database for healthy, able-bodied men and women over 70 years of age.

Altered (specific) gait pattern and lower locomotion speed are often characteristics of pathological gait observed in both sexes when compared with healthy people. It is necessary to adapt a normal gait pattern to a walking speed of people with pathological gait in reference to their sex, age, and body build. Therefore, the aim of the investigation was to establish a kinematic pattern of young female gait from motion analysis research using the BTS Smart-E system (BTS Bioengineering, Milan, Italy) conducted in the Laboratory of Biomechanical Analysis in our university. In addition to previously published data [4], this report aims at presenting gait patterns for an active, young female group for three self-selected speed levels: low (1), preferred (2), and high (3) walking speeds. This study also provides valuable comparative data, called gait patterns, for young healthy women with an average somatic body type who are within physiological norm with regard to weight-height relationship. Interindividual differences depending on age, medical history, and injuries can be observed in some gait parameters. Therefore, an understanding of normal gait patterns is needed for thorough gait patterns analysis. Gait analysis provides important information on the functional evaluation of patients and their disabilities and facilitates selection of treatment strategies.

## 2. Materials and Methods

The following mean values were collected for 20 women: age 20.14±1.18 years, body height 168.6±8.0 cm, and body mass 62.6±8.3 kg. The participants were physically fit and engaged in different types of sports and recreation activities. The women included in the study did not show obesity or excessive leanness. The group was homogeneous in terms of the type of somatic structure; it is therefore representative of the population of active women at an early age. The exclusion criteria of the study were as follows: medical history of musculoskeletal injuries causing pain, weakness, decreased range of motion, or loss of coordination, and dysfunction of the neuromuscular, cardiovascular, or respiratory systems. Anthropometric measurements were made according to International Standards for Anthropometric Assessment [17]. The following anthropometric measurements were taken: body stature (B-v), biepicondylar humerus breadth (cl-cm), biepicondylar femur breadth (epl-epm), girths of limbs, and skinfolds of trunk and limbs. Correlation between height and weight was analysed with body mass index BMI. Development of three body build components was analysed with W.H. Sheldon's technique with modifications by Heath-Carter: body-fat (endomorphy), muscle mass and bone structure development (mesomorphy), and slenderness (ectomorphy). Endomorphy was assessed with the sum of subscapular, triceps, and suprailiac skinfolds. Mesomorphy was determined on the basis of arm (biceps) and calf girths and elbow and knee breadth, while ectomorphy was verified with a slenderness ratio (HWR ratio). HWR ratio = body height/$\sqrt[{3}]{body\;\;mass}$.

Study participants were asked to walk both ways a distance of approximately 6 m with three repetitions at three different speeds: low walking speed (LWS), PWS, and high walking speed (HWS). This allowed us to isolate from six HWS cycles to eight LWS and PWS cycles for both lower extremities (studied together). The total number of gait cycles we took into account was 360 for HWS and 480 for both LWS and PWS. Participants in the first data collection were asked to walk at PWS; next the order of the tests was randomised. The instruction for LWS was “walk slowly, as if on a stroll” and for WHS “walk as fast as you can.”

The study was carried out in the certified Laboratory of Biomechanical Analysis using the BTS Smart-E system (BTS Bioengineering, Milan, Italy). The system contained six digital infrared cameras (1.1 *μ*m) at 120 Hz sampling frequency and two Network Cam AXIS 210A visible range cameras at 20 Hz frequency. A double-sided adhesive tape was used to attach the markers to the subject's body. Markers were placed above bony landmarks and wand retroreflective markers were used together with infrared illumination produced by an array of light-emitting diodes (LEDs) mounted around the lens of each camera. The standard Davis protocol (Newington model applied in the Plug-in Gait model of the BTS system) was used to determine characteristic spots on the bodies of participants [18]. Retroreflective markers were applied to the shoulders (acromion process) and to the anterior superior iliac spines (ASIS). Key spots on the lower extremities included the lateral aspect of the greater trochanter, the knee joint line (posterior to the lateral femoral condyle), the caput fibulae, lateral malleolus, the head of the fifth metatarsal bone, and the dorsum of the foot (heel bone). Attached to the pelvis was a posterior sacral marker to measure the orientation of the pelvic tilt. Two lateral wands were attached to the thigh (midway between the hip and knee joints) and the shank (midway between the knee and ankle joints). The Plug-in Gait model was used to compute three-dimensional joint angles and time–distance parameters. The following dependable variables were analysed: stride length [m], stride width [m] as the perpendicular distance between consecutive heel centres, stride time (cycle time) [s], gait speed [m/s] obtained by dividing distance travelled by cycle time, cadence [steps/sec] for whole body motion, step length [m], stance time [s], swing time [s], double stance time [s], relative stance time [% of cycle time, % CT], relative swing time [% CT], and relative double stance time [% CT] separately for both lower limbs. This was used to test gait harmony defined as the stride and time symmetry (similarity in step length and its time) [19, 20]. The symmetry index [21] was adopted to examine the differences in parameters between sides in relation to its mean values and was expressed in percentages. The following variables were determined for angular variables characterising movement at the main joints: mean value, peak positive and negative values (Peak^+^ and Peak^−^), and range of motion (ROM) calculated from the angle time characteristics. The gait cycle began when a limb first contacted the ground (at 0% GC).

For each participant, we studied 61 variables in a total of 24 gait cycles. All statistical procedures were conducted using Statistica™ 13.1 software (TIBCO Software Inc., Palo Alto, CA, USA). The minimal sample size was determined in the planning stages of the experiment using the “Power Analysis” module of the statistical software in order to get results as precisely as needed (with an assumed 5% margin of error (at 95% level of confidence), alpha level of 0.05, and accepted power of 0.80). The expected effect size was 0.57 which was needed for a moderate statistical effect. To investigate normal distribution of data in the groups we used the Shapiro-Wilk and the Kolmogorov–Smirnov tests, and The Levene's test for the analysis of variance homogeneity. All variables on both studied sides were found to be normally distributed with similar variances. The descriptive analyses of the gait variables included mean and standard deviation (SD). The dependent variables were tested to specify design with repeated measures using the analysis of variance with Bonferroni post-hoc test and compared between the LWS, PWS, and HWS speeds. The Bonferroni adjustment was preferred for matching subgroups with a smaller set of comparisons and for controlling the Type I error rate in multiple comparisons. In all tests, the statistical level of significance (*α*) was set to 0.05.

The project was approved by the university's ethical committee, and subjects gave informed consent to the work.

## 3. Results

The study group was homogenous with respect to age, body mass, and height and had similar walking patterns within speed groups. Detailed anthropometric measurements of the participants have been collected in Table 1.

The mean body height for the tested women was 168.6±8.0 cm. BMI capturing the relationship of weight and height was within the range of correct values (21.97±1.8 kg/m^2^). A low SD for the remaining features of somatic build (body breadth and width) was observed. This indicated homogeneity of the studied group in terms of body build.

The majority of spatiotemporal and angular variables showed statistical differences (*α* = 0.05) between LWS, PWS, and HWS in women (Table 2). Step length and step time proved that the gait of women was symmetrical for all speeds (symmetry index < 2%). Therefore, its characteristic was based on the right limb variables, indicating similarities between the two sides [19]. Statistically significant differences were found between LWS (1.04±0.12 m/s), PWS (1.32±0.14 m/s), and HWS (1.62±0.14 m/s). Increased walking speed had a statistically significant influence on the increase in cadence from 1.68 steps/s to 2.14 steps/s and step length from 0.56 m to 0.65 m as well as a decrease in cycle time from 1.20 s to 0.93 s. Time of individual gait cycles decreased proportionally. There was a significant decrease in the absolute stance time from 0.77 s to 0.58 s, and swing time from 0.43 s to 0.35 s. However, the relative time of these phases did not change and remained constant at around 63% CT and 37% CT for stance and swing phases.

**Figure 1:**
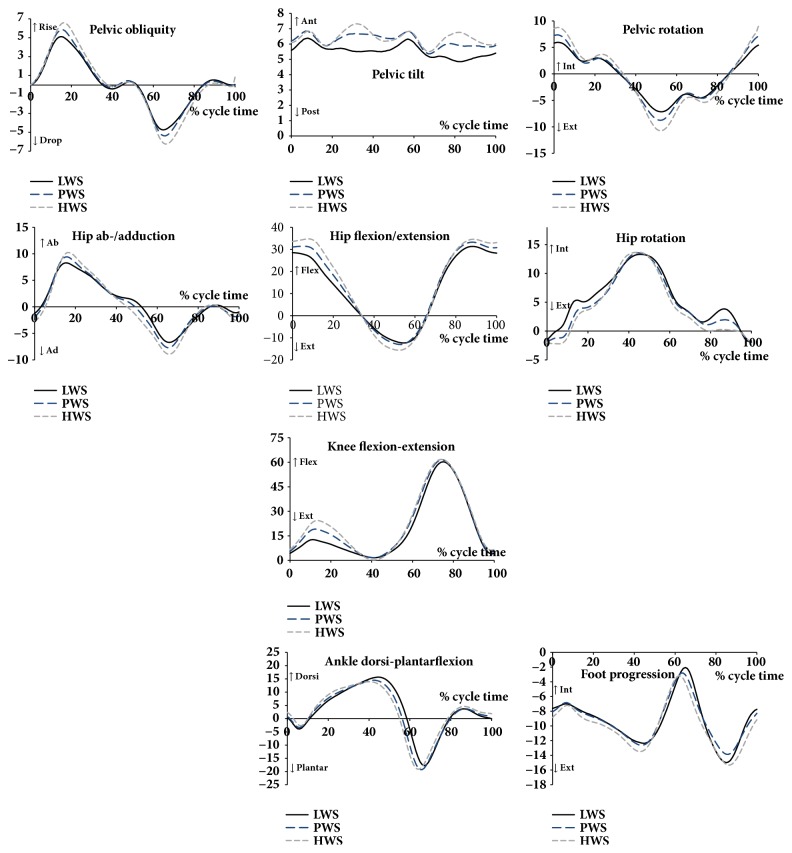
Angular kinematics for the motion of pelvis (upper row), hip (2nd row), knee (3rd row), and ankle (bottom row) joints, for the motion in frontal (left column), sagittal (middle column), and transversal (right column) planes for both sides and for the low (LWS), preferred (PWS), and high (HWS) walking speeds.

As the heel of the examined limb contacts the ground, the pelvis is nearly in neutral position (Figure 1). Shift of the weight (around 15% CT) to the trailing leg increases pelvic obliquity, since the supporting leg drops. Pelvic lift was statistically significant for HWS (6.6 deg) in comparison to LWS (5.1 deg) (Table 3). Pelvic obliquity in the swing phase was related to the drop of the contralateral limb and was statistically significant for HWS (-6.2 deg) in comparison to LWS (-4.7 deg). There were significant changes in the pelvic range of motion for HWS (12.9 deg) in comparison to LWS (9.5 deg).

In relation to neutral position, anterior pelvic tilt in the sagittal plane was 5.6 deg for LWS and 6.4 deg for HWS. The limited range of motion in this plane was around 1.5 deg and did not show any significant correlations with the speed. The results demonstrated stronger pelvic rotation in women. There were statistically significant correlations in the range of pelvic rotation with speed, which was 12.9 deg for LWS, 15.9 deg for PWS, and 19.3 deg for HWS. Pelvic movements had influence on hip movements. In the frontal plane, hip abduction occurred in the initial stance and reached a peak at 8.3 deg, 9.4 deg, and 10.2 deg (p < 0.05) for LWS, PWS, and HWS, respectively. Next, the hip adduction took place to reach a peak at -6.7 deg, -7.7 deg, and -8.9 deg for LWS, PWS, and HWS, respectively. In the frontal plane, the hip straightened at the initial phase to its peak values of -12.3 deg for LWS and -15. 6 deg for HWS (p < 0.05) and flexed to around 33 deg in the swing phase. There were statistically significant differences observed between the ROM for LWS (43.8 deg) and HWS (50.2 deg). Initial foot progression reached its peak values at about 50% CT. Foot progression peak values and ROM did not change in a statistically significant manner with the gait speed. Interestingly, one statistically significantly higher correlation was found for the mean rotation values of LWS (5.6 deg) but not for PWS (4.8 deg) or HWS (4.2 deg). The inverse effect was observed for the knee joint. As the locomotion speed increased, the knee joint flexion increased (on average 19.1 deg for LWS, 21.7 deg for PWS, and 23.1 deg for HWS). The ROM for the knee joint statistically significantly increased with the gait speed reaching 58.4 deg, 59.6 deg, and 61.3 deg for LWS, PWS, and HWS, respectively. There was a statistically significant negative relationship of the peak values of ankle dorsiflexion angle observed between the ankle joint and sagittal plane (values were 15.7 deg for LWS, 14.5 deg for PWS, and 13.9 deg for HWS), and a statistically significant correlation between the ankle plantarflexion values (-17.8 deg, -19.3 deg, and -19.2 deg, respectively) and increasing gait speed. As a result of these compensating changes, ROM remained unchanged and was around 33 deg. There were no statistically significant differences between foot progression parameters and walking speed in women.

Alterations in the gait timing for peak angular values were related to changes of individual phases in the gait model.

## 4. Discussion

The tested women represented a balanced mesoendomorphic somatotype (3.5-3.9-2.6). This means that musculoskeletal robustness and fatness were at an average and similar level. Body slenderness (ectomorphy) was the lowest in the somatotype. The results obtained can be considered typical somatic parameters for average young women [22, 23]. It is important since a number of authors have recognised that many somatic variables may have influence on gait speed and kinematic gait pattern. BMI, in particular, may influence sagittal plane pelvis motion and kinematics of hip and knee joints, while sex may differentiate the pelvic movement in the frontal plane and hip and knee joint movements [24, 25]. Age of the tested subjects is also of high relevance for gait analysis, since it may influence sagittal plane knee kinetics [24].

There are many studies that have focused on the relationship between the lower limbs and walking speed. Although our results are consistent with those obtained by different authors, they have been characterised with great detail. It is common knowledge that gait speed has significant influence on kinematic and kinetic gait patterns [4, 5]. Knee and ankle joint angular kinematic variables, in particular, show positive correlation with walking speed [26, 27]. Hanlon and Anderson [28] also indicated significant correlations between the ROM and gait speed. Our results demonstrated that speed had the greatest effect on kinematics in the knee and ankle joints (Figure 1 and Table 3). In the stance phase, peak knee flexion increased with speed, while plantar flexion occurred earlier and was greater at the end of the stance phase. Differences between the LWS, PWS, and HWS in other joints were also noticeable. Our findings are in line with previous studies [5, 29]. Fryzowicz et al. [15] demonstrated results of mean gait speed analysis that amounted to 1.37 m/s and are similar to mean speed values of the PWS (1.31 m/s) observed in our study. Female stride length (stride length 1.41 m and step length 0.64 m) and stride time (stride time 1.02 s) reported by Fryzowicz et al. [15] were similar to our findings. The lowest pelvic ROM occurred in the sagittal plane (3.6±1.1 deg) and then the frontal plane (11.5±2.6 deg) and the highest ROM was present in the transverse plane (18.0±5.6 deg). ROM for the hip joint in the sagittal plane was 50.6±4.6 deg, in the transverse plane it amounted to 22.5±5.3 deg, and in the frontal plane to 17.3±3.2 deg. Knee joint ROM in the sagittal plane amounted to 59.9±5.8 deg. The ankle ROM in the sagittal plane was 35.1±5.9 deg. Foot progression angle ROM amounted to 14.2±4.1 deg, and to 6.1±5.0 deg of external rotation in mid-stance.

The literature review shows differences in reference values for individual gait parameters, even in similar research groups. The differences may result from an application of different methods, lower or higher walking speed, or a lab environment. It should be emphasised that studies differ in data collection and measuring equipment. For instance, kinematic data of the subjects from Kuwait [9] revealed differences in gait speed between males and females for the self-selected PWS and HWS. They have shown that differences in gait patterns may result from different body build, including height and mean body mass [9–11]. Hollman et al. [16] gave reference values for a group of 1750 elderly people (70+) for 23 gait parameters, while Watelain et al. [30] differentiated kinematic gait patterns between young and elderly participants and, based on biomechanical data, introduced a classification of gait patterns for able-bodied subjects and the elderly. In particular, they showed differences between phasic and temporal parameters. Bertuit et al. [13] used the GAITRite electronic walkway to present spatiotemporal variables of gait in pregnant women compared to 23 nonpregnant participants (BMI = 22 kg/m^2^) for three different speeds, like in our study. Females in their study performed LWS (0.81±0.13 m/s) slower by 0.23m/s, PWS (1.26±0.13 m/s) slower by 0.06 m/s, and HWS (1.76±0.22 m/s) faster by 0.14 m/s in comparison to our findings. The results of that analysis compared with our results were different for cadence, step and cycle time, and swing and stance and double support ratios.

Normal gait pattern is the key component in the investigation of pathological gait patterns. This suggests that there is a need to include data from participants with different somatic structures to develop a normative database or a need to limit the database results to a specific population. A normative gait database, different than PWSs, can be used for better adjustment of reference patterns to assess locomotion disorder or pathology. Biomechanical variables can be used to classify gait patterns.

## 5. Conclusions

This study is a comprehensive analysis of gait patterns of young, healthy females with a typical body build for their population. Detailed characteristics of linear and angular kinematic variables discussed in this work allow for proper adjustment of kinematic and kinetic parameters depending on gait speed of people with locomotion disorders.

## Figures and Tables

**Table 1:** Characteristics of study participants.

	**Mean**	**SD**
Age	20.14	1.18
Body Weight (kG)	62.64	8.32
Body Height (cm)	168.61	8.03
Biepicondylar humerus breadth (cm)	6.06	0.35
Biepicondylar femur breadth (cm)	9.13	0.51
Flexed upper arm girth (cm)	28.27	3.30
Calf girth (cm)	35.83	1.72
Subscapular skinfold (mm)	9.77	2.39
Triceps skinfold (mm)	9.07	3.80
Supraspinale skinfold (mm)	15.40	5.93
Calf skinfold (mm)	5.57	2.30
Endomorphy	3.49	1.09
Mesomorphy	3.89	1.13
Ectomorphy	2.57	0.84
BMI	21.97	1.80

**Table 2:** Spatiotemporal gait variables based on the right limb kinematics for the low (LWS), preferred (PWS), and high (HWS) walking speeds. Gait phases expressed in % of cycle time (%CT) with symmetry index values.

**gait speed [m/s]**	**LWS**	**PWS**	**HWS**
	**1.04±0.12** ^∗#^	**1.32±0.14** ^∗^	**1.62±0.14**
cadence [steps/sec]	1.68±0.14^∗#^	1.94±0.1^∗^	2.14±0.1
stride length [m]	1.25±0.08^#^	1.38±0.1	1.54±0.11
stride width [m]	0.16±0.02	0.15±0.04	0.15±0.06
stride time (cycle time) [s]	1.20±0.11^∗#^	1.04±0.05^∗^	0.93±0.04
right step length [m]	0.56±0.04^∗#^	0.61±0.08	0.65±0.14
step length symmetry [%]	0.6 ± 0.2	0.4 ± 0.1	0.3 ± 0.1
stance time [s]	0.77±0.07^∗#^	0.65±0.05	0.58±0.03
relative stance time [%CT]	64.5±1.2	62.9±1.7	62.0±1.6
stance time symmetry [%]	1.1 ± 0.5	0.7 ± 0.1	0.5 ± 0.1
swing time [s]	0.43±0.04^∗#^	0.38±0.03	0.35±0.02
relative swing time [%CT]	35.5±1.2	36.6±2.1	38.0±1.7
swing time symmetry [%]	1.1 ± 0.6	0.7 ± 0.2	0.4 ± 0.1
double stance time [s]	0.17±0.03^∗#^	0.13±0.02	0.11±0.02
relative double stance time [%]	14.5±1.4	13.0±1.5	11.7±1.4
double stance time symmetry [%]	0.6 ± 0.2	0.4 ± 0.1	0.3 ± 0.1

^∗^Significant difference between LWS and PWS or between PWS and HWS, Bonferroni, p<0,05

^#^Significant difference between LWS and HWS, Bonferroni, p<0,05.

**Table 3:** Spatiotemporal gait variables: mean value, peak positive and negative values (Peak+, Peak-), and range of motion (ROM) for averaged right and left lower extremities and for the low (LWS), preferred (PWS), and high (HWS) walking speeds.

	**LWS**				**PWS**				**HWS**			
	**Mean**	**Peak** ^**+**^	**Peak** ^−^	**ROM**	**Mean**	**Peak** ^**+**^	**Peak** ^−^	**ROM**	**Mean**	**Peak** ^**+**^	**Peak** ^−^	**ROM**
**Pelvic obliquity**	0.1	5.1^#^	-4.7^#^	9.5^∗#^	0.2	5.9	-5.4	11.3	0.1	6.6	-6.2	12.9
**Pelvic tilt**	5.6^∗#^	6.4^#^	4.8	1.1	6.2	6.8^∗^	5.4	1.8	6.4	7.3	5.5	1.6
**Pelvic rotation**	-0.6	6.0^#^	-7.2^#^	12.9^∗#^	-0.6	7.4^∗^	-8.8	15.9^∗^	-0.7	9.0	-10.8	19.3
**Hip ab-adduction**	0.8	8.3^∗#^	-6.7^#^	15.3^#^	0.6^∗^	9.4	-7.7^∗^	17.0	0.2	10.2	-8.9	19.5
**Hip flexion-extension**	11.4^#^	31.4	-12.3^#^	43.8^#^	13.1	33.3	-13.0	46.4	13.7	34.8	-15.6	50.2
**Hip rotation**	5.6^∗#^	13.3	-1.8	15.1	4.8	13.6	-2.0	15.8	4.2	13.7	-2.2	15.9
**Knee flexion-extension**	19.1^∗#^	60.3	1.6	58.4^#^	21.7	61.5	1.4^∗^	59.6^∗^	23.1	61.8	0.7	61.3
**Ankle dorsi-plantarflexion**	2.8	15.7^#^	-17.8^∗#^	33.6	2.2	14.5	-19.3	33.8	2.4	13.9	-19.2	32.8
**Foot progression**	-9.3	-2.1	-15.0	12.7	-9.3	-2.8	-14.3	11.2	-10.0	-3.2	-15.3	11.8

^∗^Significant difference between LWS and PWS or between PWS and HWS, Bonferroni, p<0,05

^#^Significant difference between LWS and HWS, Bonferroni, p<0,05.

## Data Availability

The data (individual results as well as values behind the means for all measures reported and values used to build graphs) used to support the findings of this study are included within the article or are available from the corresponding author upon request.

## References

[1] Jordan K., Challis J. H., Newell K. M. (2007). Walking speed influences on gait cycle variability. *Gait & Posture*.

[2] Ralston H. J. (1958). Energy-speed relation and optimal speed during level walking. *Internationale Zeitschrift für Angewandte Physiologie Einschließlich Arbeitsphysiologie*.

[3] Sasaki K., Neptune R. R. (2006). Muscle mechanical work and elastic energy utilization during walking and running near the preferred gait transition speed. *Gait & Posture*.

[4] Pietraszewski B., Winiarski S. S., Jaroszczuk S. (2012). Three-dimensional human gait pattern - reference data for normal men. *Acta of Bioengineering and Biomechanics*.

[5] Kwon J. W., Son S. M., Lee N. K. (2015). Changes of kinematic parameters of lower extremities with gait speed: a 3D motion analysis study. *Journal of Physical Therapy Science*.

[6] Charalambous C. P. (2014). The major determinants in normal and pathological gait. *Class. Pap. Orthop*.

[7] Feng J., Wick J., Bompiani E., Aiona M. (2016). Applications of gait analysis in pediatric orthopaedics. *Current Orthopaedic Practice*.

[8] Kelch A. J., Gulgin H. R. (2017). Functional movement screen score by somatotype category. *Clinical Kinesiology*.

[9] Al-Obaidi S., Wall J. C., Al-Yaqoub A., Al-Ghanim M. (2003). Basic gait parameters: A comparison of reference data for normal subjects 20 to 29 years of age from Kuwait and Scandinavia. *Journal of Rehabilitation Research & Development*.

[10] Oberg T., Karsznia A., Oberg K. (1993). Basic gait parameters: reference data for normal subjects, 10-79 years of age. *J. Rehabil. Res. Dev*.

[11] Oberg T., Karsznia A., Oberg K. (1994). Joint angle parameters in gait: reference data for normal subjects, 10-79 years of age. *J. Rehabil. Res. Dev*.

[12] Forczek W., Staszkiewicz R. (2012). Changes of kinematic gait parameters due to pregnancy. *Acta Bioeng. Biomech*.

[13] Bertuit J., Feipel V., Rooze M. (2015). Temporal and spatial parameters of gait during pregnancy. *Acta Bioeng. Biomech. / Wroclaw Univ. Technol*.

[14] da Silva-Hamu T. C. D., Formiga C. K. M. R., Gervásio F. M., Ribeiro D. M., Christofoletti G., de França Barros J. (2013). The impact of obesity in the kinematic parameters of gait in young women. *Journal of General Internal Medicine*.

[15] Fryzowicz A., Murawa M., Kabacinski J., Rzepnicka A., Dworak L. B., Kabaciński J. (2018). Reference values of spatiotemporal parameters, joints angles, ground reaction forces, and plantar pressure distribution during normal gait in young women. *Acta Bioeng. Biomech*.

[16] Hollman J. H., McDade E. M., Petersen R. C. (2011). Normative spatiotemporal gait parameters in older adults. *Gait & Posture*.

[17] Norton K., Olds T. (2009). *Anthropometrica: a textbook of body measurement for sports and health courses*.

[18] Davis R. B., Õunpuu S., Tyburski D., Gage J. R. (1991). A gait analysis data collection and reduction technique. *Human Movement Science*.

[19] Winiarski S., Dubiel-Wuchowicz K., Rutkowska-Kucharska A. (2013). Symmetry of support scull and vertical position stability in synchronized swimming. *Acta of Bioengineering and Biomechanics*.

[20] Iosa M., Bini F., Marinozzi F. (2016). Stability and Harmony of Gait in Patients with Subacute Stroke. *Journal of Medical and Biological Engineering*.

[21] Robinson R. O., Herzog W., Nigg B. M. (1987). Use of force platform variables to quantify the effects of chiropractic manipulation on gait symmetry. *Journal of Manipulative and Physiological Therapeutics*.

[22] Galić B. S., Pavlica T., Udicki M. (2016). Somatotype characteristics of normal-weight and obese women among different metabolic subtypes. *Archives of Endocrinology and Metabolism*.

[23] Rajkumar R. V. (2015). Endomorphy dominance among non-athlete population in all the ranges of body mass index. *International Journal of Physiotherapy and Research*.

[24] Chehab E., Andriacchi T., Favre J. (2017). Speed, age, sex, and body mass index provide a rigorous basis for comparing the kinematic and kinetic profiles of the lower extremity during walking. *Journal of Biomechanics*.

[25] Harding G. T., Hubley-Kozey C. L., Dunbar M. J., Stanish W. D., Astephen Wilson J. L. (2012). Body mass index affects knee joint mechanics during gait differently with and without moderate knee osteoarthritis. *Osteoarthritis and Cartilage*.

[26] Bovi G., Rabuffetti M., Mazzoleni P., Ferrarin M. (2011). A multiple-task gait analysis approach: Kinematic, kinetic and EMG reference data for healthy young and adult subjects. *Gait & Posture*.

[27] Lelas J. L., Merriman G. J., Riley P. O., Kerrigan D. C. (2003). Predicting peak kinematic and kinetic parameters from gait speed. *Gait & Posture*.

[28] Hanlon M., Anderson R. (2006). Prediction methods to account for the effect of gait speed on lower limb angular kinematics. *Gait & Posture*.

[29] Stansfield B., Hillman S., Hazlewood M. Sagittal joint angles. moments and powers are predominantly characterised by speed of progression and not age in 7-12 years old normal children. *Journal of Pediatric Orthopaedics*.

[30] Watelain E., Barbier F., Allard P. Gait pattern classification of healthy elderly men based on biomechanical data. *Archives of Physical Medicine and Rehabilitation*.

